# Single Micrometer-Sized Gels: Unique Mechanics and Characters for Applications

**DOI:** 10.3390/gels4020029

**Published:** 2018-03-28

**Authors:** Miho Yanagisawa, Chiho Watanabe, Kei Fujiwara

**Affiliations:** 1 Department of Applied Physics, Tokyo University of Agriculture and Technology, Naka-cho 2-24-16, Koganei, Tokyo 184-8588, Japan; cwatanabe@m2.tuat.ac.jp; 2 Department of Biosciences and Informatics, Faculty of Science and Technology, Keio University, 3-14-1 Hiyoshi, Yokohama 223-8522, Japan; fujiwara@bio.keio.ac.jp

**Keywords:** janus particle, anisotropic shape, phase separation, wetting, micrometric confinement, micropipette aspiration

## Abstract

Microgels—small gels of submicron to micron size—are widely used in food, cosmetics and biomedical applications because of their biocompatibility and/or fast response to external environments. However, the properties of “single” microgels have not been characterized due to limitations in preparation technologies and measurement methods for single microgels with sizes in the multi-micrometer range. The synthesis of multiple shapes of single microgels and their characterization are important for further functionalization and application of gel-based materials. In this review, we explain the recent advancements in microgel fabrication and characterization methods for single microgels. The first topic discussed includes the self-assembly methods for single microgel fabrication using physical phenomena such as phase separation, interfacial wetting and buckling instability. The second topic deals with methods for analyzing the mechanics of single microgels and the differences between their mechanical characteristics and those of bulk gels. The recent progress in the fabrication and characterization of single microgels will bring important insights to the design and functionalization of gel-based materials.

## 1. Introduction

Polymer gels are viscoelastic materials that can store large amounts of liquid in their polymer networks. The liquid, which constitutes the majority of the polymer gel, contributes to their soft and hydrophilic properties. Because of these characteristics, polymer gels have become ubiquitous and indispensable. For example, the biocompatibility and biodegradability of hydrogels are beneficial for biomedical applications [1,2]; the controllable nature of their refractive index is applied in soft contact lenses [3] and optical lenses [4]; the functional hydrophilic material entrapped in gels is used in biomedical cosmetics and food materials [5]. Furthermore, a gel composed of a hydrophobic polymer has been featured as an oil absorbing agent for environmental recovery [6]. The growth in the versatile usage of polymer gels has prompted the necessity for improving polymer gels.

With progress in the functionalization of polymer gels, the sizes of gels have recently featured in a novel method of regulating the characteristics of gels. On account of their larger surface to volume ratios compared to bulk gels, smaller gels exhibit higher permeabilities, promoting efficient substance transport between gels and their environments [7,8]. This feature is prominent in small gels of submicron to micron size, that is, microgels. The fast response of microgels to external environments is applied in repairing damaged cell tissues [9,10,11,12,13] and the smooth release of the drugs trapped inside them [14].

The question that needs to be answered is whether a single microgel has the same characteristics as a bulk gel. Although the higher permeabilities of microgels compared to bulk gels suggests a negative answer, the elucidation of microgel properties should contribute greatly to the further functionalization of gels.

**Figure 1:**
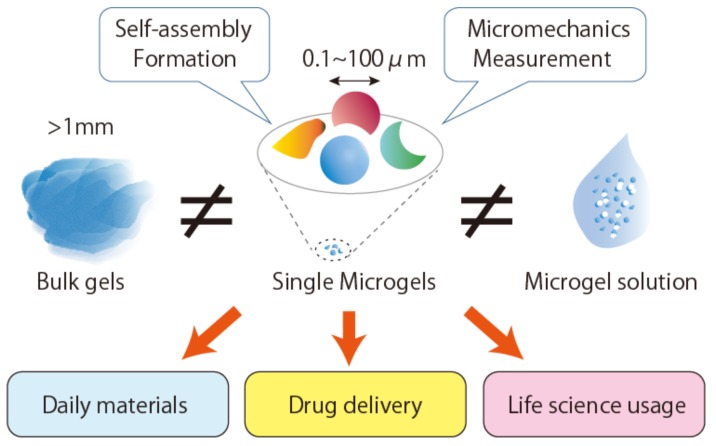
Schematic illustration of nonspherical single microgels exhibiting unique properties and applications.

Single microgels have not been characterized due to the limitations in measuring methods, unlike bulk gels and solutions containing microgels [15,16]. Recent studies have shown that single microgels exhibit unique mechanics and characters that are beneficial for numerous applications (Figure 1). For example, single microgels can generate functions according to their shapes and stabilize artificial cells like cytoskeletons in cells [17,18]. In this review, we introduce the recent advances in the preparation, characterization and application of single micrometer-sized gels using water-in-oil emulsions. Although research on nanometer-sized gels [19] is progressing and their characteristics have been analyzed using super resolution microscopy, scattering techniques and so forth. [20,21], such gels are not the focus of this review.

## 2. Morphology of Single Microgels

A major strategy for preparing microgels is the gelation of polymers inside microsized emulsions (emulsion polymerization). In general, microgels synthesized by gelation inside emulsions have spherical or core-shell shapes. Although the sphere-based shape is a common morphology for microgels, the synthesis of non-spherical particles has gained attention due to their potential for multiple applications, for example, as building blocks for complex assemblies [22,23,24], drug carriers and functional coatings [25]. Gelation inside a microsized mold is a technique to prepare non-spherical microgels. However, the preparation of molds is a laborious process. A recent study used physical phenomena such as polymer phase separation and droplet surface interaction as alternative morphology-controlling method for synthesizing non-spherical microgels [26]. This method is based on spontaneous spatiotemporal organization and therefore is expected to pave the way for forming microgels with desired morphologies without complicated molecular synthesis or expensive equipment.

### 2.1. Morphology Control through Phase Separation and Gelation of Polymers inside Microdroplets

In emulsion polymerization, the mold for gelation is usually spherical because it is the most stable shape for lipid droplets with minimum surface area. The gelation of the polymers confined in spherical droplets results in spherical microgels. The use of liquid-liquid phase separation polymers helps to obtain microgels of various shapes without designing molds. Here, we introduce a method for obtaining non-spherical shaped microgels using microdroplets (water-in-oil emulsion covered with a surfactant layer such as lipid).

**Figure 2:**
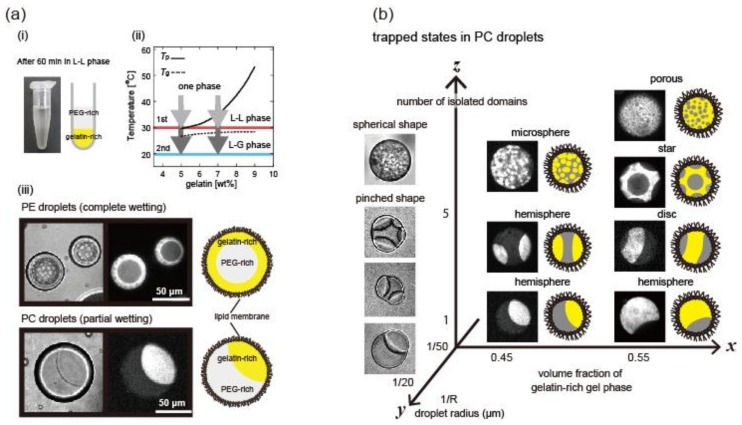
Morphology control using phase separation and gelation of gelatin inside microdroplets. (**a**) (**i**) An example of phase-separated solution of poly-(ethylene glycol) (PEG)1.7 wt % and gelatin 5.0 wt % in tube; (**ii**) Schematic phase diagram of PEG/gelatin blends containing the PEG 1.7 wt % and various concentrations of gelatin. The black solid and dashed lines indicate the phase separation point, *T*p and the gelation point, *T*g, respectively; (**iii**) Phase separation of PEG and gelatin 5.0 wt % blend in phosphatidylethanolamine (PE) and phosphatidylcholine (PC) droplets after 60 min incubation. Droplets are shown in (from left to right) differential interference contrast (DIC) images, fluorescent images of the gelatin-rich gel phase and schematic illustrations. (**b**) Variously shaped microgels in PC droplets in response to changes in volume fraction of gelatin-rich phase, droplet size and number of isolated domains. In the *x*–*z* plane, large droplets with *R* ~50 μm are shown in fluorescent images and schematic illustrations, where gelatin-rich phase is shown in white and yellow, respectively. In the *y*–*z* plane, small droplets with *R* ~ 20 μm are shown as DIC images. (M. Yanagisawa et al., Copyright 2014, National Academy of Sciences).

In the bulk (not in microdroplets), the phase separation of binary polymer solutions causes the appearance of spherical domains that minimizes the interfacial energy before the separation into two phases (Figure 2(ai)) [27]. However, spherical gels are not stable for a few reasons. The gelation space is smaller than the domain size. Since phase separation is trapped by fast gelation during the separation process, the rate of phase separation relative to gelation and/or the timing of gelation are also factors that control the shapes of microgels. The space of gelation affects the interfacial tension between the coexisting phases to alter the affinity (wettability) of the polymer on the surface of the confining space. Therefore, the phase separation and gelation of polymers inside microdroplets produce microgels of different shapes [28]. The degradation of affinity (de-wetting transition) enables the shape deformation of the phase separating droplets and their separation into two isolated droplets [29].

In this section, we explain in detail the method for producing nonspherical microgels using phase separation from the following two viewpoints: (i) gelation and wetting of polymers on the microdroplet surface; and (ii) volume fraction of coexisting phases and size of confinement space. In addition to the phase separation method, it has been shown that nonspherical microgels are formed using microfluidic devices. In this review, we do not explain the details; alternatively, we introduce review articles [30,31,32,33].

#### 2.1.1. Gelation and Wetting of Polymers on the Microdroplet Surface

Non-spherical microgels have been fabricated by the gelation of gelling-polymer-rich domains upon phase separation and wetting in microdroplets (Figure 2b) [34]. The domain morphology after the completion of liquid-liquid phase separation is determined by the wetting of the phases coexisting at the microdroplet interface. The degree of wetting is determined by the contact angle, which essentially determines the balance between the interfacial tensions existing among the coexisting phases. In the case of phase separating solutions of binary polymers inside microdroplets, the wettability is determined by the interfacial tensions between the coexisting polymers and also between the polymers and interface of the confined microdroplets. When gelatin and a poly(ethylene glycol) (PEG) solution are confined in lipid microdroplets, the wettability of gelatin depends on the type of lipid covering the droplets (Figure 2(aii)). If the two coexisting polymers have different affinities for the microdroplet interface, the shapes of the microgels will drastically change according to the characters of the interfaces, which are controlled by changing the surfactants at the interface.

**Figure 3:**
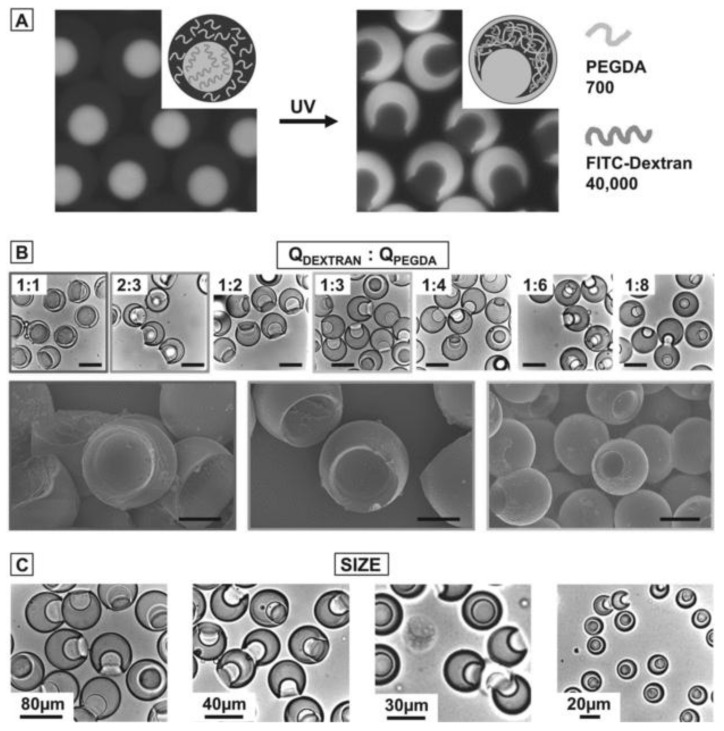
Selective polymerization of dextran/ poly-(ethylene glycol) diacrylate (PEGDA) core–shell droplets. (**A**) Fluorescence images of phase-separated aqueous two-phase system (ATPS) droplets consisting of a fluorescently labeled dextran core and a non-labeled PEGDA shell. During polymerization, dextran migrates into the PEGDA shell. As the core is depleted of dextran, the resulting microgel particle contains a socket. (**B**) Bright-field microscopy images of gel microparticles with a socket. The socket size is determined by the flow rate ratio of dextran and PEGDA (upper row). Scanning electron micrographs of selected samples (lower row). The scale bars denote 80 μm in the upper row and 30 μm in the lower row. (**C**) Bright-field microscopy images of particles with different sizes. While the overall flow rate of the ATPS mixture and the fluorinated oil is kept constant, the total particle size is determined by the flow rate ratio of the ATPS mixture and the fluorinated oil phase as well as the height of the second microfluidic nozzle, which is either 75 or 20 μm. (S. Ma et al., Copyright 2012 WILEY-VCH Verlag GmbH & Co. KGaA, Weinheim).

However, controlling the wetting property of a gelling polymer is not easy since the surface tension between coexisting phases changes before and after gelation. For example, Ma et al. reported that the phase separation pattern changes before and after UV-induced gelation of poly-(ethylene glycol) diacrylate (PEGDA) (Figure 3A) [35]. This suggests that the selective polymerization of coexisting phases alters the interfacial tension between coexisting phases and accordingly changes the wettability and contact angle. In addition, gelation increases the elasticity of gelling-polymer-rich domains. Thus, various complex patterns are formed by controlling the wetting property, especially for a system whose phase separation and gelation simultaneously progress with temperature change [36,37].

#### 2.1.2. Volume Fraction of Two Coexisting Phases and Size of the Confining Microdroplet

At a constant contact angle, changes in volume fractions can vary the shapes of single microgels (Figure 2b and Figure 3B). In the case of partial wetting, concave single microgels are formed. The crenated shape of a microgel depends on the volume fraction of the gelling-polymer rich phase [34,35]. Although such concave microgels can be formed by adding smaller particles as a mold, the advantage of the phase separation method is that it does not require mold particles [23]. The size of the microdroplet affects the microgel shape, since the balance between bending energy and interfacial energy depends on the microdroplet size (Figure 2b and Figure 3C) [35,36].

### 2.2. Morphology Control by Buckling Instability

**Figure 4:**
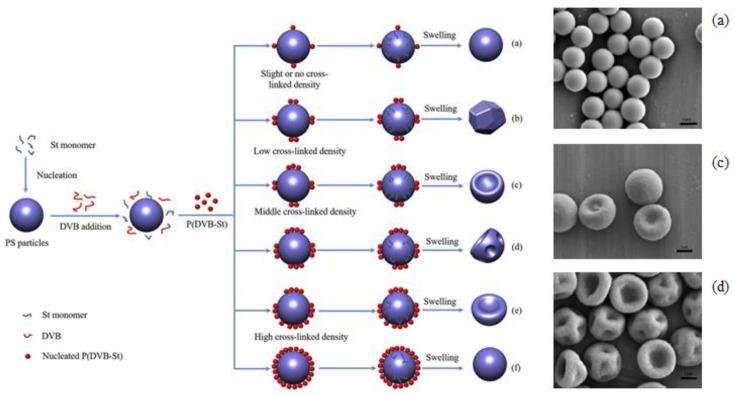
Nonspherical single microgels prepared using buckling instability. (**left**) Possible formation mechanism of the buckling of microgels with various surface morphologies; (**right**) Scanning electron microscopy (SEM) micrographs of microgels synthesized by varying the divinylbenzene (DVB) concentration based on total styrene (St) monomer mass, (**a**) 0 wt %, (**c**) 0.5 wt % and (**d**) 2.0 wt %. The scale bars are 1 μm. (H. Shen et al., Copyright 2017 ELSEVIER).

Buckling is another useful phenomenon for forming non-spherical microgels (Figure 4). Buckling instability takes place when a layer is subjected to contraction [38]. One strategy for inducing contraction is to form a heterogeneous surface gel network by adding agents that change the cross-linking gelling rate [33]. Microgels with heterogeneous crosslinks of polymer networks swell heterogeneously, leading to buckle formation. Another method is to use specially designed monomers with different cross-linking moieties [22,23,25].

Using two consecutive polymerizations, it forms soft core-hard shell structures. Consequently, mismatches between the inner and outer mechanical properties induce buckling of the surface shell. Buckled microgels can be useful for colloidal building blocks, along with smaller spherical microgels [24].

## 3. Micromechanics Measurement of Single Microgels

**Figure 5:**
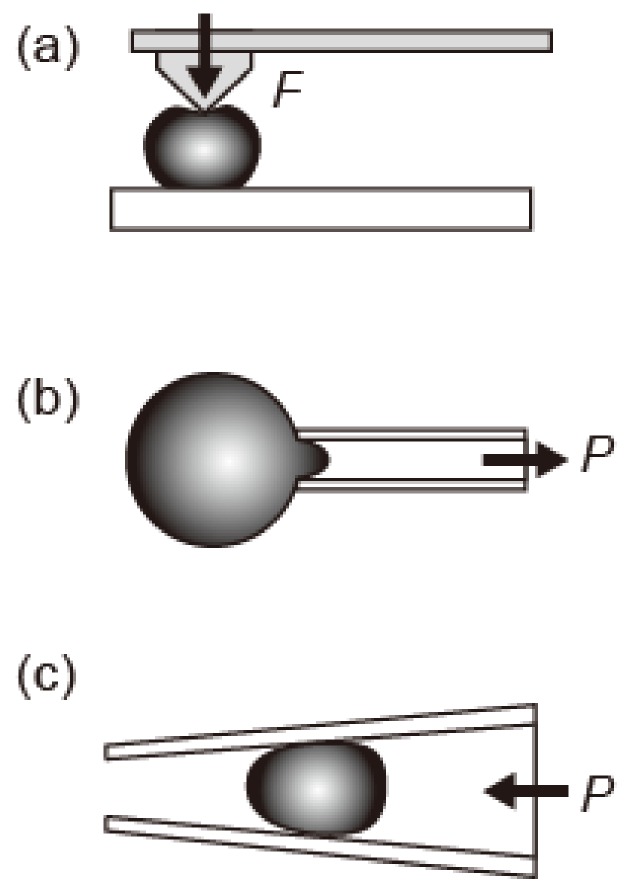
Schematic illustrations of methods for measuring the elastic properties of single microgels. (**a**) Atomic force microscopy (AFM) and (**b**,**c**) microcapillary. The single microgel is (**b**) aspirated inside the microcapillary and (**c**) deformed inside the microcapillary. The F and P represent the applied force by AFM tip and applied pressure by microcapillary, respectively.

Microgels are soft and deformable microparticles. As mentioned above, it is important to elucidate the mechanical properties of single microgels rather than their solution for application purposes. However, in contrast to the accumulation of the macroscopic mechanical properties of microgel solutions, the microscopic mechanical properties of single microgels were ambiguous. Experimental difficulty was the obstacle in analyzing the microscopic mechanical properties of a single microgel. To solve the problem, methods involving atomic force microscopy (AFM) [39,40,41] and microcapillary methods for analyzing the deformation during suction [42,43,44,45,46] and compression [42,47,48] have recently been employed (Figure 5).

In the case of AFM measurement, two technical difficulties remain: placing soft particles on a flat substrate and avoiding the deformation of the particle during pressing by AFM tips for measurement (Figure 5a). Recently, Mohapatra et al. reported that the induced Young’s modulus of an air-dried microgel obtained using AFM was different from that obtained using Brillouin light scattering (BLA) (Table 1) [40]. Such AFM measurements become more difficult in the case of microgels floating in a liquid. In contrast, when using microcapillaries, it is easy to measure the microgels floating in a liquid, since aspirating or trapping a single microgel in the capillary can be used to measure the elasticity (Figure 5b,c) [42]. In the following sections, we introduce the microscopic mechanical properties of single microgels, in particular their elasticity as revealed by the microcapillary method.

### 3.1. Micromechanics of Single Microgels

In the emulsion polymerization method, the physicochemical environments around the monomers of the gels near the surface are different from that in other areas. Therefore, the balance between the rate of surface polymerization and diffusion of the polymerization field across the droplet is considered a factor determining its mechanical properties. In fact, the balance has been reported to change the shell thicknesses of core-shell microgels [2,49], which is an important parameter determining their mechanistic properties. For instance, the mechanical stability of a single microgel increases as shell thickness increases [50] and the elasticity varies depending on the size ratio of the core-to-shell thickness [48,49]. Although the correlation between the mechanical properties and structure of microgel is not yet clear, these reports demonstrate that the kinetics of gelation is important in determining the mechanical properties of single microgels.

**Figure 6:**
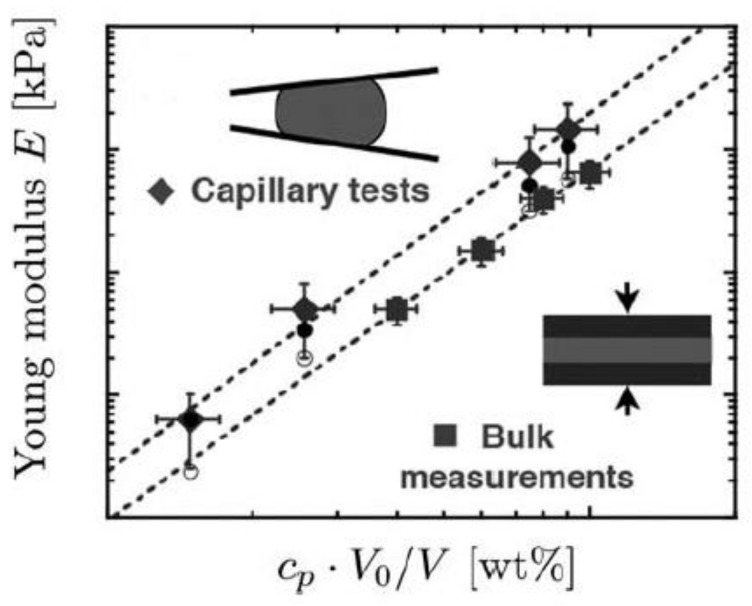
Young’s modulus *E* from compressive mechanical tests on bulk polyacrylamide gels (squares) and for microgel particles (diamonds) as derived from the compressive modulus *K* (solid circles) and the shear modulus *G* (open circles). To account for the swelling of the particles in water, the polymer concentration *c*p is corrected by the volume ratio *V*0/*V* (*V*0 is the initial volume of the particles as they are formed in oil and *V* is the equilibrium volume of the collected particles in water). (H. M. Wyss et al., Copyright 2010 Royal Society of Chemistry).

In general, microgels with high surface areas/volume ratios exhibit immediate responses to changes in external environments and alter their structures and mechanical properties to fit their environments. In fact, the elasticity of the poly(*n*-isopropylacrylamide) (PNIPAM) microgel has been reported to change greatly depending on the surrounding environment (temperature, solvent, etc.) [51]. In addition, it was reported that the Young’s modulus of PNIPAM microgel is larger than that of the corresponding bulk gel (Figure 6) [47]. Furthermore, Young’s moduli of polystyrene-*co*-poly(*n*-isopropylacrylamide) (pS-*co*-NIPAM) microgels are sensitive to the cross-link concentration, unlike the bulk modulus (Table 1) [40].

Recently, our group measured the elasticities of gelatin single microgels prepared inside microdroplets covered with lipid layers by micropipette aspiration [46]. We found that the elasticities of small microgels with radius less than 50 μm are higher than those of the corresponding bulk gels. In this study, the structural changes in gelatin molecules induced by the lipid membranes covering the microdroplets were pointed out as a factor for the increase in elasticity.

In addition, in non-spherical microgels (Figure 2 and Figure 3), only a part of the microgel is in contact with the surfactant film, such as a lipid layer covering the droplet. Therefore, the shapes and surface dynamics of non-spherical microgels are expected to be anisotropic. Since microcapillary aspiration (Figure 5b) can measure the local elasticity of the microgel, the correlation between shape and local elasticity will be revealed in the near future.

As described above, the characteristics of lipid membranes may change the surface gel structure and the mechanical properties of the gels due to lipid membrane-polymer interactions. These effects should be the case for biological single microgels like cytoskeletons underneath cellular membranes. Thus, elucidation of the unique characters of single microgels and their mechanisms should contribute to understanding the nature of living cells.

### 3.2. Structural Differences between Single Microgels and Bulk Gels

Why does a single microgel show gel elasticity different from that of the bulk gel? This is because gel elasticity is determined by the polymer network structure constituting the gel and the difference in gel elasticity indicates that the micro or nanostructures of microgels are different from those of bulk gels.

In the case of emulsion polymerization, gelation starts from the droplet surface of gelling polymers. A balance between the gelation and penetration rates of the cross-linking agent for gelation into the interior possibly renders the network structure of the microgels heterogeneous. This heterogeneous microgel structure could be the reason for the buckling of microgels (Figure 4) [40]. If this is the case, the surfactant film covering the droplet surface changes the interaction between the surface and the gelling polymer encapsulated inside. This effect will also result in a different microgel structure compared to the bulk gel. We recently found that the microgel elasticity is higher than that of the bulk gel as a result of changing its secondary structure by synthesizing a gelatin gel in lipid-film-covered microdroplets [46].

Biopolymers such as gelatin are known to correlate with lipids and similar materials in cell membranes [52]. Therefore, this progress indicates that it is possible to control the structures and elasticities of microgels via the correlation between the surfactant membrane covering the microdroplet surface and the gelling polymer. Since reducing the size of the microgel increases the ratio of surface area to internal volume, elastic control of microgels through size is also expected.

## 4. Summary and Perspective

Without complicating the molecular design of gelling polymers, it is now possible to change the shape of single microgels by utilizing phase separation between the non-gelling polymer and the gelling polymer inside microdroplets. By developing new methods to measure the elasticity of a single microgel, the unique mechanical properties of microgels (different from those of bulk gels) have been revealed. Further elucidation of the structure of the microgel and the process of gelation and functionalization utilizing the shape and mechanical properties of the microgel are expected in the near future.

## Figures and Tables

**Table 1:** Comparison of Young modulus determined for the air-dried polystyrene-co-poly(*n*-isopropylacrylamide) (pS-*co*-NIPAM) microgel series with different cross-link concentration (represented by numbers following the name) using Brillouin light scattering (BLA) and AFM. (H. Mohapatra et al., Copyright 2017 Royal Society of Chemistry).

Microgel Sample	Bulk Modulus, *K* (GPa)	BLS Young’s Modulus, *E* (GPa)	AFM Young’s Modulus, *E* (GPa)
pS-*co*-NIPAM-1	4.92 ± 0.2	3.59 ± 0.14	0.24 ± 0.03
pS-*co*-NIPAM-2	4.86 ± 0.19	3.70 ± 0.15	0.54 ± 0.03
pS-*co*-NIPAM-3	4.86 ± 0.19	4.07 ± 0.16	0.61 ± 0.05
pS-*co*-NIPAM-4	4.92 ± 0.2	4.25 ± 0.16	0.91 ± 0.06
pS-*co*-NIPAM-5	5.02 ± 0.2	4.65 ± 0.19	0.99 ± 0.03
